# Personality Traits of the Territorial Crustacean Chinese Mitten Crab (*Eriocheir sinensis*): Behavioral Adaptations to Environmental Cues

**DOI:** 10.3390/ani15050757

**Published:** 2025-03-06

**Authors:** Peiqiong Fang, Sisi Sheng, Yiming Li, Yuan Li, Renhua Mo, Han Mei, Guangzhen Jiang, Wenbin Liu, Hengtong Liu

**Affiliations:** Key Laboratory of Aquatic Nutrition and Feed Science of Jiangsu Province, National Experimental Teaching Center for Animal Science, College of Animal Science and Technology, Nanjing Agricultural University, No. 1 Weigang Road, Nanjing 210095, China; m13486068681@163.com (P.F.); 15371917570@163.com (S.S.); 18912973962@163.com (Y.L.); lyscjdmn@163.com (Y.L.); 15677441687@163.com (R.M.); 18372898090@163.com (H.M.); jianggz@njau.edu.cn (G.J.); wbliu@njau.edu.cn (W.L.)

**Keywords:** boldness, aggression, behavioral adaptation, environmental cues, *Eriocheir sinensis*

## Abstract

Territorial crustaceans exhibit a complex set of personality traits and behavioral characteristics that are critical for their survival, growth, and overall welfare. This study aimed to systematically investigate the interplay between two essential personality traits—boldness and aggression—in Chinese mitten crabs (*Eriocheir sinensis*), during various developmental stages under conditions of crowding stress and food odor stimuli. Using behavioral assays, we found that adults displayed significantly higher levels of boldness and aggression compared to juveniles. Notably, bold adult crabs demonstrated lower aggression than their shy counterparts, revealing a significant negative correlation between boldness and aggression under crowding stress. Furthermore, juvenile and adult crabs exhibited negative correlations between the stability of their boldness and aggression with individual levels of boldness and aggression, respectively. The study also found that while juvenile boldness did not significantly correlate with body size, larger individuals displayed higher boldness under stress, contrasting with adult crabs, where boldness negatively correlated with body size, although aggression levels were unaffected by size; the stability of adult boldness demonstrated a positive correlation with body size under crowding stress. These findings elucidate the intricate dynamics of personality traits in territorial crustaceans, laying the groundwork for future research into their adaptive behavioral strategies in response to environmental cues like group stress and food odors.

## Simple Summary

In the context of behavioral research on territorial crustaceans, the personality traits of reared animals have attracted much attention. Crowding stress and food odors are two prevalent environmental factors that influence animal behavior, yet their impacts on the formation of animal personalities remains largely unexplored. In this study, we assessed the personality traits (boldness and aggression), and the plasticity thereof, of the Chinese mitten crab (Eriocheir sinensis) when exposed to crowding stress and food odors. Our findings indicated higher levels of boldness and aggression in adult crabs compared to younger crabs. The boldness and aggression of adult crabs were negatively correlated under crowding stress. The plasticity of boldness in adult crabs was more sensitive to food odors, whereas their aggression plasticity was more sensitive to crowding stress. The boldness of young crabs was positively correlated to their morphological characteristics, while in adults these two factors were negatively correlated. Aggression of adult crabs was positively correlated with morphological characteristics under the effect of food odors. These discoveries imply adaptive behavioral tactics with reference to personality traits and their plasticity in territorial crustaceans subjected to crowding stress and food odors in rearing environments.

## 1. Introduction

Animal personality refers to a set of specific persistent behavioral characteristics of individual animals and stable behavioral differences between individuals over time and/or situations [1]. Recent evidence showed that more than 10% of the variation in animal behavior components is related to their “personality”, including social behavior, territorial behavior, reproductive behavior, learning behavior, feeding behavior, etc. [2]. Over the past two decades, research on animal personality has expanded, primarily focusing on vertebrates such as reptiles [3], birds [4], rodents [5] and other mammals [6]. In aquatic animals, relevant research mainly targets fish [2,7,8], while studies on crustaceans remain scarce. Boldness and aggression are two personality traits that have received considerable attention in animal personality research, as they shape both inter- and intraspecific interactions [9,10,11]. Boldness is the tendency of individuals to explore unfamiliar environments and is fundamental to animal personality research [12]. Aggression, on the other hand, is an important component of inter- and intraspecies competition, crucial for obtaining resources such as mates, food, and territory, and is often closely linked to other behaviors, such as boldness [13]. For crustaceans, boldness and aggression are closely related to their initiative to explore the environment, motor activity, and intra- and inter-species confrontation capabilities. Together, these factors directly affect their feeding efficiency and survival, especially in high-density farming conditions. Recently, Su et al. [14] demonstrated that bold swimming crabs (*Portunus trituberculatus*) exhibit higher feeding efficiency than their timid counterparts. Subsequently, Zhang et al. [7] confirmed that boldness affects the aggressive behavior in swimming crabs, with bold individuals showing greater willingness and ability to attack than shy ones. Liang et al. [15] further evidenced that, when environmental resources (including food and space) are scarce, bold swimming crabs tend to look for new resources, while aggressive individuals are more effective at competing for available resources, thus improving their survival in challenging environments. These previous studies highlighted the practical significance of personality traits like boldness and aggression in crustaceans to boost aquaculture production. However, studies on other economically important crustaceans are significantly lacking, and the complex link between these two personality traits remains unrevealed.

Considering personality plasticity, extensive studies suggested that animal personality behaviors are adaptable and influenced by environmental conditions [15]. While individual behavior may remain stable in a consistent environment, it can change significantly when environmental factors shift. In intensive farming systems, the crowding stress from high breeding density and food-related odors are key environmental cues that influence individual behavior. Researchers found in their studies that overcrowding is a significant source of stress for zebrafish (*Danio rerio*) causing anxiety and behavioral changes, in which the bold individuals could maintain their bravery and display heightened aggression in crowded situations and stronger predatory competition [16,17,18]. Similarly, a study by Axling et al. [13] revealed that aggressive Atlantic salmon (*Salmo salar* L.) individuals became more aggressive when crowding stress increased. While existing research demonstrated that crowding stress plays a significant role in shaping the personality behaviors of aquatic animals, studies on crustaceans remain limited. Meanwhile, recent studies have explored how environmental olfactory signals relate to animal personality behavior. Aquatic animals mainly rely on sensitive chemical sensory organs in complex underwater environments to detect various odor signals, thereby generating directed behavioral responses critical for feeding, survival, and reproduction [7]. For example, crayfish (*Procambarus clarkii*) keenly use their olfactory system to accurately identify odor sources, monitor predator presence, and adjust their behavioral strategies for finding shelter and food [19]. In a study of Mexican cavefish (*Astyanax mexicanus*), Blin et al. [20] found that individuals with varying personality traits exhibited different levels of odor sensitivity and tracking abilities, and these odor signals also influenced the behavior strategies of individual cavefish. However, research is lacking on the interaction between food odor signals and personality performance of farmed animals in aquaculture environments, particularly in crustaceans known for their high territorial awareness. In summary, research examining the relationship between environmental signals—such as crowding stress and food odors—and animal personality behaviors is expected to reveal the behavioral adaptability mechanisms of farmed animals in high-density farming systems and provide fundamental data for optimizing aquacultural practices and animal welfare.

To investigate the adaptation of personality behaviors driven by environmental cues in a crustacean model, we employed Chinese mitten crab (*Eriocheir sinensis*), commonly referred to as river crab. This species is widespread in the continental shelf and freshwater regions along the Bohai Sea, Yellow Sea and East China Sea, representing both economic and ecological significance. Currently, the main breeding method for the Chinese mitten crab is pond breeding, where the injuries and deaths resulting from their inherent territorial behavior and aggression seriously affect the yield and quality of this species. However, no studies have reported the effects of crowding stress and food odor stimulation on the personality behavioral phenotypes of the Chinese mitten crab. Here, we focused on the Chinese mitten crab, recorded and characterized the boldness and aggression behaviors of individuals at various development stages using behavioral methods, analyzed the relationship between these traits, and explored their stability under different crowding- and food-related environmental signals. The aims of the study are to reveal the personality behavior patterns of Chinese mitten crab and their strategies adaptation to the potential environmental factors influencing behavior performance, which provide a crucial basis for enhancing the breeding and management practices of economically significant crustaceans.

## 2. Materials and Methods

### 2.1. Ethics Statement

All experimental protocols were granted approval by the Animal Care and Use Committee of Nanjing Agricultural University (Permit number: SYXK (Su) 2021-0086). The experimental operations involving animals were performed in adherence to the Guidelines for the Care and Use of Laboratory Animals in China.

### 2.2. Animal Rearing and Experimental Setup

Juvenile male crabs (weighing 14.50 ± 9.89 g, collected in June 2023) and adult male crabs (weighing 96.88 ± 40.97 g, collected in September 2023) were obtained from the aquaculture base of Nanjing Agricultural University (China). All crabs used had intact claws and appendages and were subsequently transferred to a controlled laboratory. All the adult animals used in the experiment were from the same batch of crab seedlings and were of the same age when they were collected. At the same time, the carapace width of the adult crabs we used ranged from 52.8 mm to 65.4 mm, ensuring that they had completed the same number of molts. Crabs were maintained at 28 ± 0.5 °C with a 12 h:12 h light–dark cycle, and a dim light of 200 lux was consistently used during the lighting period to avoid causing stress to the animals due to excessive brightness. Crabs were acclimated in separate PVC tanks (40 cm in diameter, 60 cm in height) for 7 days and were fed commercial feed pellets once daily. The leftover feed and feces were removed after 2 h of feeding, and the 1/3 of the water was changed. The serial number of each crab was marked on the carapace using acrylic paint for subsequent tracking of individual behavior in the experimental setup (described below).

### 2.3. Overview of Experimental Design

**Figure 1:**
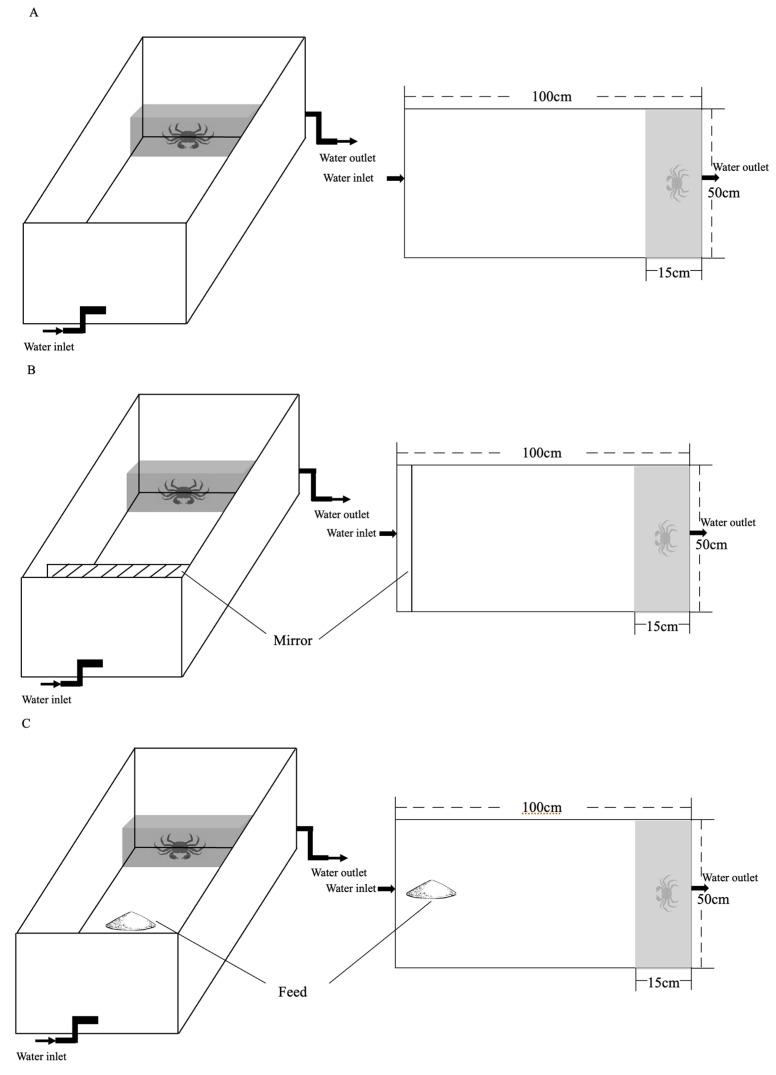
Personality behavior observation setup. (**A**) Boldness and aggression behavior observation setup in IP group, (**B**) boldness and aggression behavior observation setup under crowding stress (SP group), and (**C**) boldness and aggression behavior observation setup under food odor stimulation (FP group).

To test and characterize the individual personality behavioral phenotypes of boldness and aggression in Chinese mitten crab, we subjected 30 juveniles and 30 adults to three sequential tests (Figure 1)—an inherent personality behavior test under control conditions (refer to as IP group), a personality behavior test under crowding stress conditions (referred to as SP group), and a test under food odor stimulus group (referred to as FP group). One end of the experimental tank was connected to the water inlet pipe as the upstream end and the other end was connected to the outlet pipe as the downstream, with a consistent water velocity of 0.1 m/s. In the behavior test under crowding stress conditions, a mirror (50 cm × 25 cm) fixed on the end of the observation zone was employed to create a visual cognition of increased population density, and thus to mimic a crowding stress environment. The purpose of setting up a mirror was to simulate the presence of additional crabs, enhancing the perceived density within the enclosure. To avoid excessive stress responses that affect the normal behavior of animals, we just set up a mirror in the box. In the behavior test under food odor stimulation, 10 ± 1 g of commercial feed pellets was stored in a perforated pipe located near the upstream water inlet pipe, allowing the odors of food to reach the crabs with the current. At the end of each test, the crabs were then returned to their home tanks to rest for three days and were fasted for 12 h before the next test in order to standardize hunger level.

The experimental setup consisted of an observation zone (PVC tank of 100 cm × 50 cm × 45 cm, length × width × height) and an infrared HD photography system (1080p camera, Hikvision, Hangzhou, China) fixed 1 m above observation zone (as shown in Figure 1). During each test, boldness and aggression traits were successively assessed. For each trial, crabs were removed in groups of five from their housing tanks using hand netting, and placed within a black box (50 cm × 15 cm × 10 cm) positioned on the downstream end of the observation tank for a 10 min acclimatization to new environment. After the acclimation, once the sliding door of the black box was opened, allowing crabs to roam freely, the test and a 10 min video shooting began. We extracted two widely used tracking data measures to characterize individuals’ boldness: the latency to emerge from the initial black shelter and the total time spent outside the shelter. Based on boldness behavior in other taxa, our prediction is that bolder individuals will tend to emerge from shelter more quickly and spend a longer time exposed in the open area. We considered that the crab had emerged from the shelter when its entire carapace passed over the sliding door. In the aggression test, we extracted two traits to assess individuals’ aggression levels: the frequency of active aggression (individual crab first raises its claws to attack other crabs) and the duration of individual fights (the total duration of active and passive aggression by an individual crab during the test period, Table 1). All the trials were carried out between 09:00 and 13:00, with individuals tested in a random order. On any given day, all trials conducted in the laboratory were of a single assay type. These experimental tanks were filled with 10 cm of oxygenated water with >5.0 mg/L dissolved oxygen and pH 7.0–8.0. The water was changed after each trial to avoid any influence of conspecific cues that might change animal’s behavior. After behavioral tests, all crabs were individually measured in terms of their body weight, as well as the width, length and height of their carapace, as essential indicators of body size.

### 2.4. Statistical Analysis

Statistical analyses were performed in R version 4.4.1/R Commander Package. All recorded behavioral acts were converted to a proportion of the total time of the test (600 s). Data were presented as means ± standard deviation. To evaluate individual boldness and aggression by combining multiple behavioral traits and compare between three experimental groups, we ran a Principal Component Analysis (PCA), using a table providing the behavioral traits data for all individuals in the three groups. We then retrieved the scores for individuals on the first axis of the PCA, and used these coordinates as a synthetic indicator of the individual score of boldness and aggression for a certain experimental condition. According to the retrieved scores from high to low, we clustered all the tested crabs as bold (top 1/3), medium, and shy (bottom 1/3). The relationship between plasticity of personality behavior and individual inherent personality traits were also estimated using the same linear model, where the behavioral variations were calculated as the differences between boldness and aggression levels in two treatment groups and the control group. The regression model was considered significant at *p* < 0.05 level. The relationship between individual boldness and aggression, as well as the relationship of the level of boldness and aggression and individual morphological indicators (including body weight and body size) were estimated by a simple linear regression model.

## 3. Results

### 3.1. Comparative Analysis of Boldness and Aggression of Chinese Mitten Crab at Different Developmental Stages

**Figure 2:**
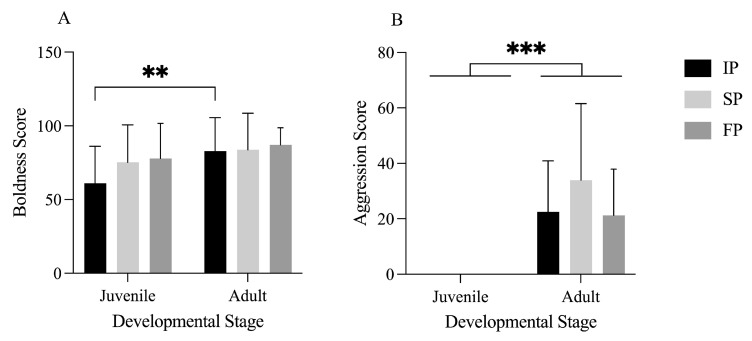
Comparative analysis of the boldness and aggression of juvenile and adult Chinese mitten crabs. (**A**) is a comparative analysis of the boldness of juvenile and adult crabs, and (**B**) is a comparative analysis of the aggression of juvenile and adult crabs. ** and *** indicate *p* < 0.01, and *p* < 0.001, respectively.

The personality behavior profiling was conducted in three groups: the control group (initial personality, IP), the crowding stress stimulation group (crowding stress-induced personality, SP), and the food odor stimulation group (food odor-induced personality, FP). In the IP group, adult crabs demonstrated significantly greater boldness than juvenile crabs (*p* < 0.05). Crowding stress and food odor slightly increased the boldness of both juvenile and adult crabs, but this change was not statistically significant (Figure 2A). In all three experimental groups, adult crabs exhibited significantly greater aggression than juvenile crabs (*p* < 0.05). Crowding stress (SP), rather than food odor cues, had a slight but noteworthy impact on the aggression of adult crabs, although this effect was not significant (Figure 2B).

### 3.2. Analysis on the Relationship Between Boldness and Aggression of Chinese Mitten Crab

**Figure 3:**
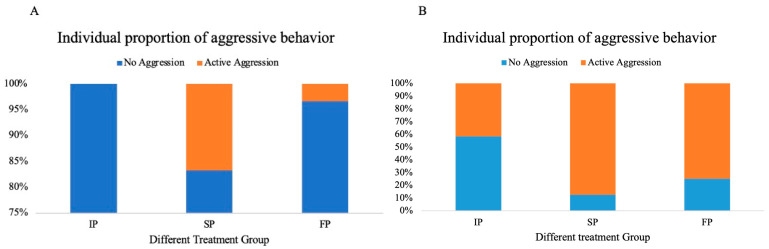
The proportion of aggressive behaviors in each group of juvenile (**A**) and adult (**B**) crabs.

**Figure 4:**
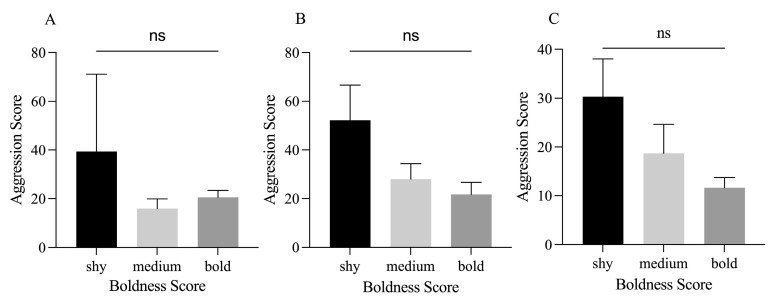
Comparative analysis of aggression of adult crabs with different levels of boldness; (**A**) is the relationship between boldness and aggression of adult crabs, (**B**) is the relationship between boldness and aggression of adult crabs under population pressure, and (**C**) is the relationship between boldness and aggression of adult crabs under feed odor stimulation. Data are shown as mean ± SD (*n* = 30); ns, not significant (Kruskal–Wallis test).

**Figure 5:**
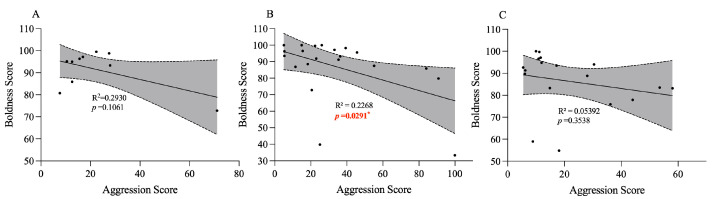
Regression analysis of boldness and aggression in adult crabs. (**A**) is the regression analysis of boldness and aggression in the IP group of adult crabs that showed aggressive behavior; (**B**) is the regression analysis of boldness and aggression in the SP group of adult crabs that showed aggressive behavior, and (**C**) is the regression analysis of boldness and aggression in the FP group of adult crabs that showed aggressive behavior. The gray areas in the figure represent confidence intervals, the black dots represent individual personality ratings, and * represents *p* < 0.05.

Under our experimental conditions, juvenile crabs exhibited a low level of aggression. The proportion of juvenile crabs initiating active attacks in the IP, SP, and FP groups accounted for 0%, 16.7%, and 3.3%, respectively; while the proportions for adults were 41.7%, 87.5%, and 75% in the respective groups (Figure 3). Given the low level of aggression exhibited in juvenile crabs, this study focused on adult crabs to examine the relationship between boldness and aggression traits. An interesting trend of lower aggression in bold adult crabs compared to their shy counterparts was observed in all three groups, although this difference was not significant (Figure 4). A significant negative relationship was identified between individual boldness and aggression scores in the SP group (*p* = 0.0291, Figure 5B), whereas the IP and FP groups displayed non-significant negative correlations (Figure 5A,C), indicating that crowding stress, rather than food odor stimulation, could significantly influence the relationship between boldness and aggression personality traits in adult crabs.

### 3.3. Analysis of the Behavioral Plasticity of Personality Traits in Chinese Mitten Crab

**Figure 6:**
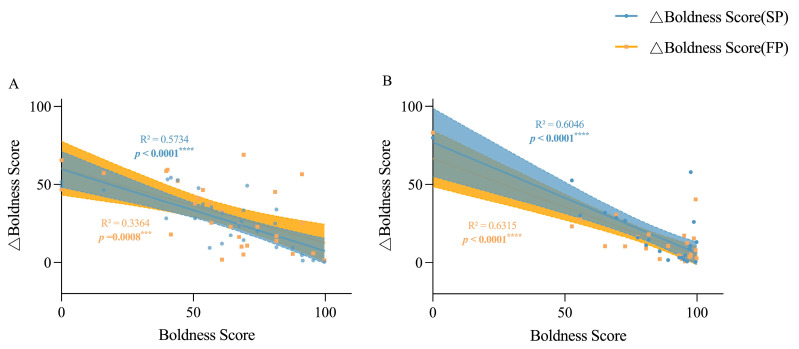
Linear relationships between the boldness score and the boldness score change value in the Chinese mitten crab at different developmental stages. (**A**) is the linear relationship between boldness score and boldness score change value of juvenile crabs, and (**B**) is the linear relationship between the boldness score and the boldness score change value of adult crabs. Boldness score change value = |individual boldness score − boldness score under population pressure/food odor stimulation|; this means that △Boldness Score = |Boldness Score (IP) − Boldness Score (SP/FP)|. The yellow area represents the confidence interval between the bravery score and the bravery score difference of group SP, and the blue area represents the confidence interval between the bravery score and the bravery score difference of group FP. *** means *p* < 0.001, **** means *p* < 0.0001, The same goes for the figures below.

**Figure 7:**
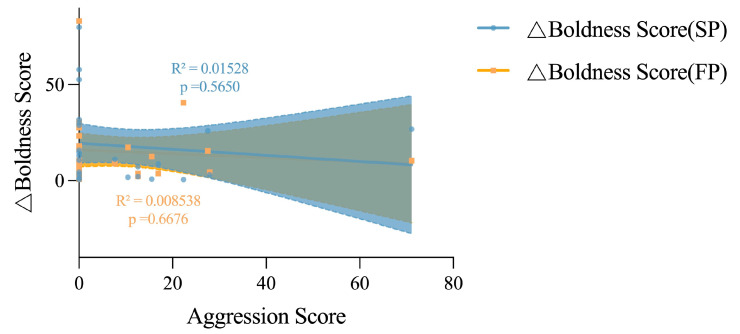
Linear relationships between the change value of boldness score and aggression score of adult crabs. The yellow area represents the confidence interval between the bravery score and the bravery score difference of group SP, and the blue area represents the confidence interval between the bravery score and the bravery score difference of group FP.

**Figure 8:**
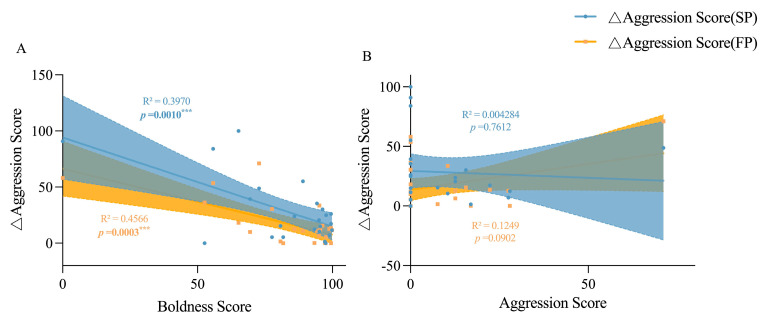
Linear relationships between the change value of adult crab aggression score and its boldness score and aggression score. (**A**) is the linear relationship between the boldness score of adult crabs and the change value of the aggression score, and (**B**) is the linear relationship between the aggression score of adult crabs and the change value of aggression score. Change values of aggression = |individual aggression score − population pressure/aggression score under food odor stimulation|; that is, △Aggression Score = |Aggression Score (IP) − Aggression Score (SP/FP)|. *** indicate *p* < 0.001.

The variation in boldness levels for both juvenile and adult crabs in the SP and FP groups decreased as their boldness levels increased, compared to the IP group (*p* < 0.0001 and *p* = 0.0008 in juvenile crabs; and *p* < 0.0001 and *p* < 0.0001 in adult crabs, respectively, as seen in Figure 6), suggesting that bold individuals are generally more consistent in their boldness than their shy counterparts. Meanwhile, juvenile crabs exhibited greater boldness plasticity in response to food odor stimulation than to crowding stress (*p* < 0.0001), indicating a behavioral strategy that crabs adopt a braver and more exploratory act when encountering food cues and peer threats, with the former being of greater significance. There was no significant correlation between aggression and boldness in adult crabs (Figure 7). The variation in aggression levels for adult crabs in the SP and FP groups decreased more as their boldness levels increased compared to the IP group (*p* = 0.0010 and *p* = 0.0003, respectively), wherein crowding stress may have had a stronger impact on the individual aggression plasticity (Figure 8). In adult crabs, the variation in boldness was positively correlated with the level of individual aggression in the SP group, but negatively correlated in the FP group. Together, these findings revealed an environmentally driven relationship between the personality behavioral plasticity and individual’s inherent personality traits, in which bolder and more aggressive individual crabs tend to be more stable in their boldness and aggression behavior performance when encountering crowding stress and food availability issues; meanwhile, food availability has a stronger impact on individuals’ boldness, while crowding stress has a stronger impact on individuals’ aggression.

### 3.4. The Involvement of Body Size in Shaping Personality Behavioral Strategies

**Figure 9:**
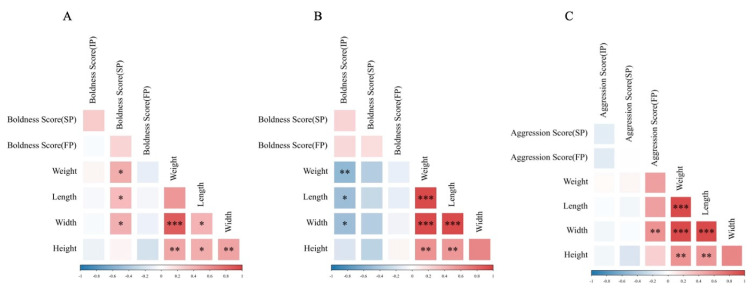
The heat map of the correlation between personality and morphological characteristics of Chinese mitten crab. (**A**) is the heat map of juvenile boldness score and morphological characteristics, (**B**) is the heat map of adult boldness score and morphological characteristics, and (**C**) is the heat map of adult aggression score and morphological characteristics. *, **, and *** indicate *p* < 0.05, *p* < 0.01, and *p* < 0.001, respectively.

**Figure 10:**
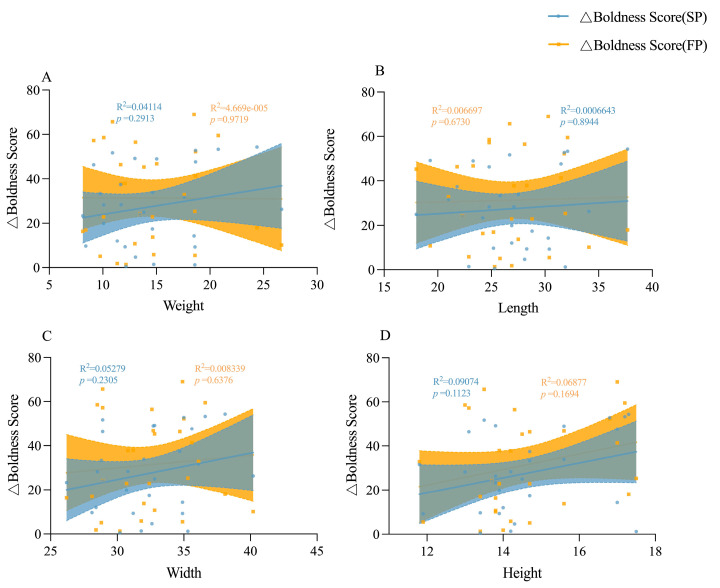
Linear relationships between the boldness score change value and morphological characteristics of juvenile crabs. (**A**) is the linear relationship between the boldness score change value and weight of juvenile crabs, (**B**) is the linear relationship between the boldness score change value and body length of juvenile crabs, (**C**) is the linear relationship between the boldness score change value and body width of juvenile crabs, and (**D**) is the linear relationship between the boldness score change value and body height of juvenile crabs.

**Figure 11:**
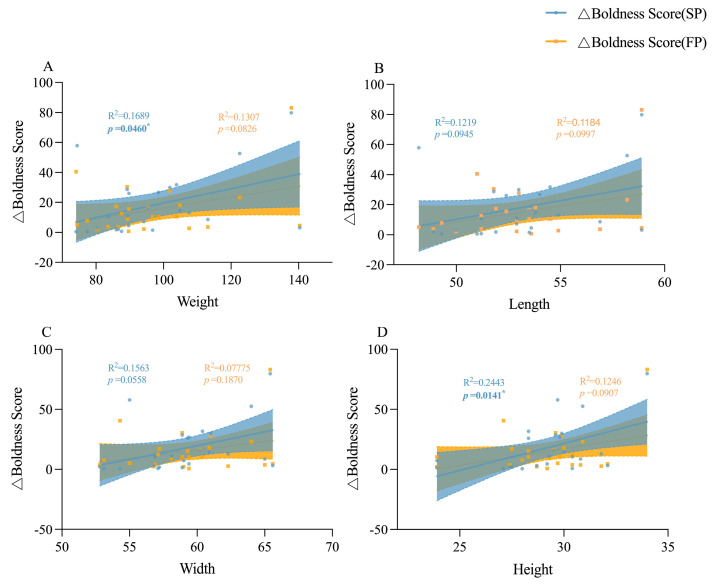
Linear relationships between the boldness score change values and morphological characteristics of adult crabs. (**A**) is the linear relationship between the boldness score change value and weight of adult crabs, (**B**) is the linear relationship between the boldness score change value and body length of adult crabs, (**C**) is the linear relationship between the boldness score change value and body width of adult crabs, and (**D**) is the linear relationship between the boldness score change value and body height of adult crabs. * indicates *p* < 0.05.

**Figure 12:**
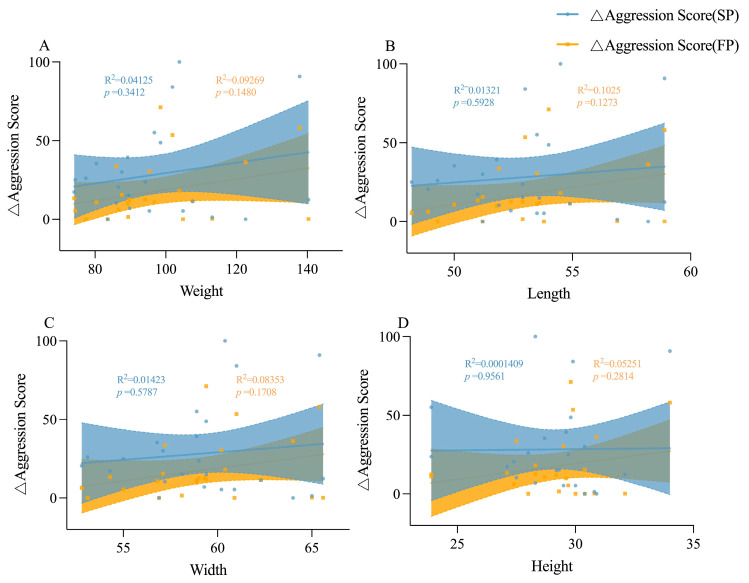
Linear relationships between the aggression score change values and morphological characteristics of adult crabs. (**A**) is the linear relationship between the aggression score change value and weight of adult crabs, (**B**) is the linear relationship between the aggression score change value and body length of adult crabs, (**C**) is the linear relationship between the aggression score change value and body width of adult crabs, and (**D**) is the linear relationship between the aggression score change value and body height of adult crabs.

Under crowding stress conditions, juvenile boldness score was positively correlated with body weight, body length and body width (*p* < 0.05, Figure 9A); adult boldness score was negatively correlated with body weight, body length and body width (*p* < 0.05, Figure 9B), and adult aggression score was positively correlated with body width (*p* < 0.01) when stimulated by food odor (Figure 9C). Under crowding stress and food odor, no significant relationship was found between boldness variation and morphological characteristics in juvenile crabs (Figure 10). However, in adult crabs, boldness variations exhibited a positive correlation with their morphological characteristics when stimulated by food odor. In particular, there was a significant positive correlation with body weight (*p* < 0.05, Figure 11 and Figure 12). These observations indicated a positive correlation between the variations in personality traits (boldness) and body size indicators in adult crabs, where larger individuals tend to adopt a radical behavior strategy characterized by increased boldness when food is available. In contrast, body size might not influence the personality behavioral strategies in juvenile crabs.

## 4. Discussion

Boldness and aggression are considered two key behavioral traits reflecting individual personality. Boldness is generally defined as a coping strategy individuals use when facing threats or challenges, while aggression refers to the extent of an individual’s response to external stimuli or intraspecific competitors. These two traits play important roles in the environmental adaptation and behavioral strategy shifts in crustaceans, thereby affecting their success in resource competition, predation, growth, and reproduction. Numerous studies have revealed how crustaceans’ personality traits are related to their developmental stages [15,21,22]. A personality study on *Kryptolebias marmoratus* revealed that average boldness increases with age—shy individuals become bolder, and bold individuals sustain their boldness. These differences in personality expression within a developmental framework were found to be linked to metabolic rate and risk perception of environmental stressors [23]. Research on swimming crab (*Portunus trituberculatus*) similarly found that individual personality behaviors evolve with developmental stages, with juvenile crabs exhibiting lower levels of boldness and aggression than adult crabs [15]. Our study shows that adult crabs exhibit greater boldness and aggression compared to juvenile crabs. This finding may be related to the different energy states of adult and juvenile crabs, where adult crabs typically have a superior energy state and stronger territorial awareness than juveniles. Consequently, they are more likely to explore unknown territories and compete for limited space and food resources. In this study, we also found that when crabs experience crowding stress, their aggression tends to increase, regardless of the developmental stages. However, Liu et al. [24] discovered that increased population density of swimming crabs led to a decrease in aggression among adult crabs. Bell et al. [25] found that in three-spined stickleback (*Gasterosteus aculeatus*), as the individual grew, the population density gradually increased, and the lack of food and space resources also led to similar changes in their personality, including decreased aggression. These findings contradict our results, which may be due to the innate differences in behavior between species or variations in experimental conditions and population density tested. In our study, we used visual crowding stress rather than physical crowding stress, which may lead to different observations regarding changes in aggression. And these individuals lived in a pond environment, and the experience of each was different, which may have led to personality changes due to different environmental factors.

In recent years, numerous studies have examined the relationship between boldness and aggression in aquatic animals, particularly in the context of breeding production. Fu et al. [26] revealed a positive correlation between the boldness of Black Rockfish (*Sebastes schlegelii*) and their aggression levels. Similar findings were reported in personality studies of swimming crab (*Portunus trituberculatus*) [7,15] and Killifish (*Rivulus hartii*) [8]. However, Queller et al. [27] found that bold individuals of Swordtail fish (*Xiphophorus nigrensis*) exhibit lower levels of aggression in complex environments. Thus, the relationship between individual boldness and aggression is complex and may be influenced by various factors, including species, developmental stages, and environmental conditions. This complexity drives researchers to explore this issue further. Our study found no significant correlation between boldness and aggression in adult crabs. However, when crowding stress increased, individual boldness was significantly negatively correlated with aggression. This finding indicates that adult crabs may not adopt high aggression when they perceive crowding stress. Instead, they may tend to avoid conflict and adopt more conservative behavior strategies. In contrast, adult crabs did not exhibit the same conservative behavior patterns when exposed to food odor signals, suggesting that crowding stress has a greater impact on the relationship between boldness and aggression than food signals do. Overall, these findings highlight the importance of considering demographic influences on individual behavior in studies of crustaceans and territorial fish. 

It is worth noting that current reports on crustacean personality are limited, and little is known about the occurrence patterns of personality traits and the underlying behavioral strategies in complex environments. The relevant research has the potential to provide valuable insights into for feeding strategies, growth optimization, and enhancing breeding welfare. Together, we believe that the relationship between crustacean boldness and aggression is more complex than previously reported, and it may closely relate to individual behavioral motivation and environmental adaptability. In crowded living spaces, individuals may adopt conservative forms of low aggression and higher courage in response to threats in order to improve their chances of survival and feeding. However, the exact implications of this mechanism and its ecological significance require further study to better understand how economic crustaceans adapt their behavior in various ecological environments.

Studies have shown that the behavioral personality performance of aquatic animals is susceptible to environmental changes and exhibit notable plasticity. When faced with diverse environmental conditions, an individual animal adjusts its behavioral patterns to better adapt to the living environment [28,29]. This plasticity in behavioral traits is reflected in various aspects, including predation strategies, social behavior, and escape response [30,31]. Su et al. [14] revealed that feeding strategies significantly affect the aggressive and boldness behavior of swimming crabs, wherein short-term food reduction leads to increased attack frequency, benefiting bolder individuals; conversely, long-term food reduction results in fewer attacks. In the present study, we discovered that environmental factors play essential roles in shaping the behavioral personality phenotype of Chinese mitten crabs. Adult crabs exhibit less-stable personalities than juveniles when exposed to crowding stress and food odors. This could be due to either the adult crabs’ superior risk coping abilities or their greater sensitivity to environmental signals, both of which warrant further investigation. Meanwhile, we found that bolder individuals showed less fluctuations in their boldness and aggression when encountering crowding stress and food odors, indicating that bold individuals may be more conservative in modifying their personality behavior. It is worth noting that food odor exposure might cause greater fluctuations in individual boldness, while crowding stress might cause greater fluctuations in aggression. These observations suggest that individual animals may choose to make a behavioral trade-off after considering their energy status, metabolic level, and activity ability. This choice can lead to better foraging opportunities and help them protect their living space and resources, ultimately creating a behavioral response mechanism driven by various environmental signals [32]. The findings of this study provide evidence for behavioral trade-offs and adaptive decision-making selection behaviors in Chinese mitten crabs, which account for the noticeable behavioral differences among individuals in the cultured group. It is crucial to note that plasticities in behavioral syndromes among a population are not the result of a random evolutionary process, but rather are driven by the adaptive evolution of behavior, which selects the most advantageous combination of behavioral traits. Investigating the personality traits and flexibility of territorial crustaceans is crucial for finding effective ways to mitigate economic losses due to their aggression in intensive aquaculture.

Recently, the link between individual personality behavior and body size in territorial crustaceans has attracted increasing attention. Research on hermit crabs (*Pagurus bernhardus*) has shown that larger individuals exhibited lower boldness when experiencing disturbances [21,33]. It evidenced a negative correlation between body size and both movement vitality and boldness in crayfish (*Cherax destructor*); similarly, Lord [34] demonstrated that body size also influences attack behavior in juvenile American lobsters (*Homarus americanus*), with smaller individuals attacking more frequently for territorial competition, while larger individuals exhibit shorter attack durations, and the duration of their attacks is shorter than that of larger lobsters. These and other related studies have further emphasized the effect of body size on individual personality behaviors. Our study found a positive correlation between juvenile body size and their boldness only under crowding stress, but not under food odor conditions or in the control setting. In adult crabs, we observed that boldness decreased as body size increased, while under crowding stress conditions and food cues, larger and shyer individuals displayed increased boldness; meanwhile, individual aggression did not correlate with body size, yet, when food was present, larger crabs showed increased aggression, which was positively related to their size. In addition, body size is often a reflection of age in crustaceans, as larger individuals typically undergo more molts and are older. Among juvenile crabs, larger individuals are typically older and exhibit behaviors that align more closely with adults. Taking all of the above into consideration, our observations suggest that when sensing crowding stress and food odors, crabs evaluate their body size and available resources, and broaden their activity space within the range allowed by their body size advantages to explore unknown resources. For both young and adult individuals, body size may be an important limiting factor in enhancing their boldness and aggression behaviors. In crowded living environments with limited food, larger crabs can better mobilize their physical responses to compete for habitat and food. These findings establish a crucial foundation for understanding the link between individual personality behavior and morphological characteristics of crustaceans. Body size metrics are expected to become important measurable indicators of crustacean personality traits. However, the adaptive behavioral strategies and physiological mechanisms linking individual body size and personality phenotypes still need to be further investigated. Future studies should aim to clarify the behavioral ecology of economically important crustaceans by exploring these mechanisms, thereby establishing a stronger scientific basis for their aquaculture management and welfare measures.

## 5. Conclusions

Our study offered insights into how boldness and aggression in territorial crustaceans relate to their behavioral adaptations to environmental cues, such as crowding stress and food odors. First, adult crabs were bolder and more aggressive than juveniles, indicating that adults may be more inclined to explore unknown areas and compete for limited space and food resources. Second, bold adult crabs exhibited lower aggression levels than shy individuals. Under crowding stress conditions, boldness and aggression were negatively correlated, suggesting that individuals may balance their boldness and aggression traits, while, even under stress, crabs would avoid reckless actions such as exposing themselves to danger or aggressively challenging others. Additionally, when bolder individuals faced crowding stress and food availability, their boldness and aggression traits remained more stable. This suggested that bold individuals may be more conservative in modifying their behavioral traits. Finally, in juvenile and adult crabs, individuals with bigger sizes were able to increase their boldness and aggression under crowding stress conditions and food odor exposure, in contrast to the smaller ones, demonstrating that crabs may weigh their own body size and available resources when under stress, allowing them to expand their activity area to find new resources. Body size may limit how crabs can enhance their personality traits: larger individuals may be better equipped to mobilize their physiological capabilities to compete for limited resources. These findings contribute to our understanding of the personality behavioral syndromes of territorial crustaceans and their wisdom in behavioral adaptations to environmental cues.

## Figures and Tables

**Table 1:** Behavioral traits recorded for assessment of boldness and aggression indicators.

Personality Component	Behavior Phenotype	Definition
Boldness	Latency to emerge from the initial black shelter	The time when crab first crawled out of the shelter after the experiment started.
Boldness	Total time spent outside the shelter	Total time that crab spent outside the black shelter during the entire 10 min experiment.
Aggression	The frequency of active aggression	Individual crab first raises its claws to attack other crabs.
Aggression	The duration of individual fights	The total duration of active and passive aggression by an individual crab during the test period.

## Data Availability

The data presented in this study are available in the article. Further information is available upon request from the corresponding author.

## References

[1] Bridger D., Bonner S.J., Briffa M. (2015). Individual quality and personality: Bolder males are less fecund in the hermit crab *Pagurus bernhardus*. Proc. R. Soc. B Biol. Sci..

[2] Whittaker B.A., Consuegra S., de Leaniz C.G. (2021). Personality profiling may help select better cleaner fish for sea-lice control in salmon farming. Appl. Anim. Behav. Sci..

[3] Kudo H., Nishizawa H., Uchida K., Sato K. (2021). Boldness–exploration behavioral syndrome in wild sub-adult green sea turtles caught at Oita, Japan. Appl. Anim. Behav. Sci..

[4] Richardson K.M., Parlato E.H., Walker L.K., Parker K.A., Ewen J.G., Armstrong D.P. (2019). Links between personality, early natal nutrition and survival of a threatened bird. Philos. Trans. R. Soc. B.

[5] Forkosh O. (2021). Animal behavior and animal personality from a non-human perspective: Getting help from the machine. Patterns.

[6] Santicchia F., Wauters L.A., Dantzer B., Westrick S.E., Ferrari N., Romeo C., Palme R., Preatoni D.G., Martinoli A. (2020). Relationships between personality traits and the physiological stress response in a wild mammal. Curr. Zool..

[7] Zhang Z., Lin W., He D., Wu Q., Cai C., Chen H., Shang Y., Zhang X. (2023). Aquaculture environment changes fish behavioral adaptability directly or indirectly through personality traits: A case study. Rev. Fish Biol. Fish..

[8] Chen Y., Li W., Xiang L., Mi X., Duan M., Wu C. (2022). Fish personality affects their exposure to microplastics. Ecotoxicol. Environ. Saf..

[9] Wolf M., Van Doorn G.S., Leimar O., Weissing F.J. (2007). Life-history trade-offs favour the evolution of animal personalities. Nature.

[10] Shi Y., Yue Y., Shi C., Wang J., Zhang Y. (2024). Personality shapes inter-and intraspecific interactions in rodents: A behavioral experiment with Brandt’s voles (*Lasiopodomys brandtii*). Anim. Biol..

[11] Mitchell D.J., Beckmann C., Biro P.A. (2024). Maintenance of behavioral variation under predation risk: Effects on personality, plasticity, and predictability. Am. Nat..

[12] Zhu B., Su X., Yu W., Wang F. (2022). What forms, maintains, and changes the boldness of swimming crabs (*Portunus trituberculatus*)?. Animals.

[13] Axling J., Vossen L.E., Peterson E., Winberg S. (2023). Boldness, activity, and aggression: Insights from a large-scale study in Baltic salmon (*Salmo salar* L.). PLoS ONE.

[14] Su X., Zhu B., Wang F. (2022). Feeding strategy changes boldness and agonistic behaviour in the swimming crab (*Portunus trituberculatus*). Aquac. Res..

[15] Liang Q., Su X., Wang F., Zhu B., He M. (2020). The developmental plasticity of boldness and aggressiveness in juvenile and adult swimming crab (*Portunus trituberculatus*). Front. Mar. Sci..

[16] de Abreu M.S., Demin K.A., Amstislavskaya T.G., Strekalova T., Kalueff A.V. (2021). Zebrafish Models for Stress Research. Stress: Genetics, Epigenetics and Genomics.

[17] Roy T., Bhat A. (2018). Repeatability in boldness and aggression among wild zebrafish (*Danio rerio*) from two differing predation and flow regimes. J. Comp. Psychol..

[18] Parker M., Hillman C., Fontana B., Amstislavskaya T., Gorbunova M., Altenhofen S., Barthelson K., Bastos L., Borba J., Bonan C. (2024). Housing and Husbandry Factors Affecting Zebrafish (Danio rerio) Novel Tank Test Responses: A Global Multi-Laboratory Study. https://www.researchsquare.com/article/rs-4849877/v1.

[19] Ramberg-Pihl N.C., Yurewicz K.L. (2020). Behavioral responses of northern crayfish (*Faxonius virilis*) to conspecific alarm cues and predator cues from smallmouth bass (*Micropterus dolomieu*). Mar. Freshw. Behav. Physiol..

[20] Blin M., Valay L., Kuratko M., Pavie M., Rétaux S. (2024). The evolution of olfactory sensitivity, preferences, and behavioral responses in Mexican cavefish is influenced by fish personality. Elife.

[21] Briffa M., Archer R. (2023). Size specific boldness associated with differences in resource requirements and habitat use: A cross-sectional study in hermit crabs. Curr. Zool..

[22] Cabrera D., Nilsson J.R., Griffen B.D. (2021). The development of animal personality across ontogeny: A cross-species review. Anim. Behav..

[23] Edenbrow M., Croft D. (2013). Environmental and genetic effects shape the development of personality traits in the mangrove killifish *Kryptolebias marmoratus*. Oikos.

[24] Liu D., Wang F., Lu Y., Zhu B., Zhang H. (2022). Effects of stocking density on a typical crab-clam polyculture system: Behavioral mechanisms of predation and competition in swimming crab (*Portunus trituberculatus*). Aquaculture.

[25] Bell A.M., Sih A. (2007). Exposure to predation generates personality in threespined sticklebacks (*Gasterosteus aculeatus*). Ecol. Lett..

[26] Fu Y., Zhang Z., Zhang Z., Shen F., Xu X., Li Z., Zhang Y., Zhang X. (2021). Boldness predicts aggressiveness, metabolism, and activity in black rockfish *Sebastes schlegelii*. Front. Mar. Sci..

[27] Queller P.S., Shirali Y., Wallace K.J., DeAngelis R.S., Yurt V., Reding L.P., Cummings M.E. (2022). Complex sexual-social environments produce high boldness and low aggression behavioral syndromes. Front. Ecol. Evol..

[28] Rickward R.A. (2024). The Causes and Consequences of Personality Variation in the Red Cherry Shrimp, *Neocaridina Heteropoda*. Master’s Thesis.

[29] Carli G., Farabollini F. (2022). Defensive responses in invertebrates: Evolutionary and neural aspects. Prog. Brain Res..

[30] Salerno C.M., Kamel S.J. (2023). Behavioural type, plasticity and predictability are linked to shell shape in a marsh ecosystem predator–prey interaction. Anim. Behav..

[31] Fernö A., Folkedal O., Nilsson J., Kristiansen T.S. (2020). Inside the fish brain: Cognition, learning and consciousness. Welf. Fish.

[32] Su L., Lu L., Si M., Ding J., Li C. (2024). Effect of Population Density on Personality of Crayfish (*Procambarus clarkii*). Animals.

[33] Ferderer A., Davis A.R., Wong M.Y. (2022). Temperature and body size influence personality and behavioural syndromes in an invasive crayfish. Anim. Behav..

[34] Lord J.P. (2021). Size affects intraspecific aggression and response to predation threat in juvenile American lobsters. Mar. Biol..

